# Seasonal Effects of Habitat on Sources and Rates of Snowshoe Hare Predation in Alaskan Boreal Forests

**DOI:** 10.1371/journal.pone.0143543

**Published:** 2015-12-30

**Authors:** Dashiell Feierabend, Knut Kielland

**Affiliations:** 1 Department of Biology and Wildlife, University of Alaska Fairbanks, Fairbanks, Alaska, United States of America; 2 Institute of Arctic Biology, University of Alaska Fairbanks, Fairbanks, Alaska, United States of America; University of Sydney, AUSTRALIA

## Abstract

Survival and predation of snowshoe hares (*Lepus americanus*) has been widely studied, yet there has been little quantification of the changes in vulnerability of hares to specific predators that may result from seasonal changes in vegetation and cover. We investigated survival and causes of mortalities of snowshoe hares during the late increase, peak, and decline of a population in interior Alaska. From June 2008 to May 2012, we radio-tagged 288 adult and older juvenile hares in early successional and black spruce (*Picea mariana*) forests and, using known-fate methods in program MARK, evaluated 85 survival models that included variables for sex, age, and body condition of hares, as well as trapping site, month, season, year, snowfall, snow depth, and air temperature. We compared the models using Akaike’s information criterion with correction for small sample size. Model results indicated that month, capture site, and body condition were the most important variables in explaining survival rates. Survival was highest in July, and more generally during summer, when alternative prey was available to predators of hares. Low survival rates coincided with molting periods, breeding activity in the spring, and the introduction of juveniles to the sample population in the fall. We identified predation as the cause of mortality in 86% of hare deaths. When the source of predation could be determined, hares were killed more often by goshawks (*Accipiter gentilis*) than other predators in early successional forest (30%), and more often by lynx (*Lynx canadensis*) than other predators in black spruce forest (31%). Great horned owls (*Bubo virginianus*) and coyotes (*Canis latrans*) represented smaller proportions of hare predation, and non-predatory causes were a minor source (3%) of mortality. Because hares rely on vegetative cover for concealment from predators, we measured cover in predation sites and habitats that the hares occupied and concluded that habitat type had a greater influence on the sources of predation than the amount of cover in any given location within a habitat. Our observations illustrate the vulnerability of hares to predators in even the densest coniferous habitat available in the boreal forest, and indicate strong seasonal changes in the rates and sources of predation.

## Introduction

Snowshoe hares (*Lepus americanus*) are the primary prey of numerous mammalian and avian predators in the boreal forests of North America. Most juvenile and adult hares die from predation [1], so their survival depends largely on avoiding predators by occupying or remaining near dense vegetative cover [2–5] and by limiting movement [6, 7]. The amount of vegetative cover available to hares can influence both the risk of mortality [8] and sources of predation [9–11], and appears to play a larger role in habitat selection than plant species composition or food availability [1, 12–14]. However, hares in northern regions move among a wide range of vegetation communities over diel and seasonal time scales in their search for food and mates while also retaining access to escape cover [15, 16]. These shifts in habitat use, along with seasonal changes in deciduous foliage and snow cover, are likely to affect sources and rates of hare mortality. Sources of mortality are also likely to change annually for hares in northern populations where regular cycles in hare abundance and survival [17–19] are linked with functional and numerical responses exhibited by their predators [20–22].

Vegetation mediates hare survival not only by acting as a physical barrier between hares and their predators, but also by indirectly affecting a hare’s body condition via food availability, foraging movements, and microclimate. Body condition is likely to influence survival most during winter when a hare’s diet is restricted to low-quality woody browse. This may be especially true of hares in interior Alaska and Canada where extended periods of extreme cold regularly expose hares to temperatures well below their thermoneutral zone [23]. In this geographic region, there is often a marked contrast in the browse and vegetative cover available to hares in commonly used habitats, such as in young deciduous and dense coniferous forests. Hares appear to be faced with a tradeoff between predation risk and food quality; in this case, choosing between the dense vegetative cover but relatively poor browse commonly found in coniferous forests, and the sparse cover but relative abundance of select foods typically found in young deciduous forests. Our study addressed the potential effects of this apparent tradeoff on hare survival and predation, across seasonal and annual time scales, by monitoring radio-tagged individuals inhabiting a mosaic of boreal forest communities in interior Alaska.

Snowshoe hare ecology has been studied extensively in the Kluane region of the Yukon Territory, Canada, where the majority of snowshoe hare studies in the northern boreal forest have taken place, yet the coniferous forests of interior Alaska often differ markedly from those in the Kluane region with respect to species composition and structure [24, 25]. The biological and climatic differences among these regions warrant further investigation into the interactions among snowshoe hares, competing herbivores, and hare predators. Our study contributes to a better understanding of North American boreal forest ecology by investigating the seasonality of habitat-specific survival and predation rates, and the wide range of factors governing these rates, for snowshoe hares in Alaska.

This study took place during the late-increase, peak, and initial decline phase of a population cycle; a time when hares are expected to occupy dense vegetation communities such as black spruce (*Picea mariana*) forests and disperse into habitats with less vegetative cover such as young deciduous forests [15, 26, 27]. We identified sources of mortality for radio-tagged hares and, in instances of predation, related habitat and vegetative cover characteristics of kill sites to predator class and species. We then used known-fate survival models to identify the importance of habitat, body condition, age, sex, and environmental parameters to hare survival over monthly, seasonal, and annual periods. To our knowledge, this is the first study to incorporate such a variety of variables into survival models for snowshoe hares over a large portion of the population cycle.

Based on previous work that suggested a tradeoff between understory cover and food availability [4, 12, 15], we hypothesized that survival and mortality sources would exhibit pronounced spatial and temporal patterns. Deciduous forest should provide higher quality food to hares than coniferous forest throughout the year, but should lack sufficient cover when deciduous leaves are absent, whereas coniferous forest should provide hares with considerable cover in all seasons. Therefore, we predicted that (1) snowshoe hare survival would be higher in deciduous rather than coniferous forest during the summer when leaves provided high quality food and additional cover from predators, but that survival would be higher in coniferous forest during other times of the year; (2) both mammalian and avian predation rates would be highest in deciduous forest when deciduous leaves were absent, but would be constant across seasons in coniferous forest; and (3) both mammalian and avian predation would occur in sites with lower than average understory cover, and avian predation would occur in sites with lower than average canopy cover as well. We also predicted that survival would be (4) lower for juveniles than adults due to inexperience with, and selection by, predators [9, 28, 29]; (5) lower for males than females due to higher rates of movement by males [30], especially around times of mating; and (6) lower for hares in poorer body condition due to depleted energy reserves available for thermoregulation and predator escape [31].

## Study Area

**Fig 1:**
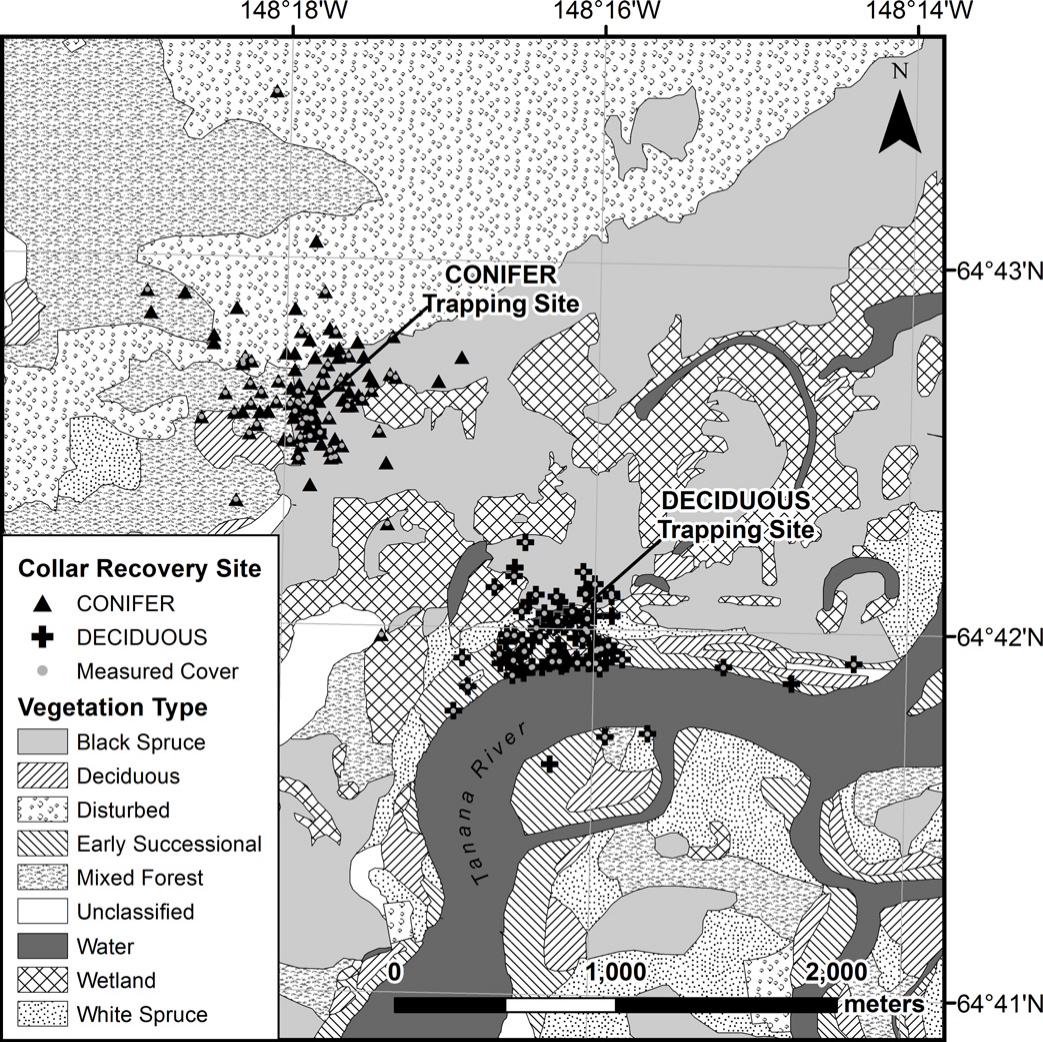
Trapping sites and VHF collar recovery locations for snowshoe hares. Hares were collared in Bonanza Creek Experimental Forest near Fairbanks, Alaska, from June 2008 to May 2012. Figure includes data previously published in Feierabend and Kielland [16].

This study took place in the Bonanza Creek Experimental Forest (64° N, 148° W), located approximately 20 km southwest of Fairbanks, Alaska (Fig 1). This area consists of a mosaic of floodplain, lowland, and upland vegetation types that include early successional forest, balsam poplar (*Populus balsamifera*), white spruce (*Picea glauca*), black spruce, muskeg, wetland, mixed forest, shrub birch (*Betula* spp.), Alaska birch (*Betula neoalaskana*), aspen (*Populus tremuloides*), and recently burned communities. Snowshoe hare population dynamics have been monitored here since 1998 [32]. We used established trapping grids in two representative snowshoe hare habitats (hereafter referred to as “Deciduous” and “Conifer”) with populations sufficiently large for estimating hare densities and survival. The Deciduous grid was in an early successional community dominated by willow (*Salix* spp.), thin-leaf alder (*Alnus tenuifolia*), and balsam poplar, located adjacent to the Tanana River. Understory species included *Epilobium angustifolium*, *Cornus canadensis*, *Calamagrostis canadensis*, and *Equisetum* spp. The Conifer grid was in a mature black spruce community with an understory composed of *Ledum* spp., *Rosa acicularis*, *Vaccinium vitis-idaea*, *Salix* spp., *Chamaedaphne calyculata*, mosses, and lichens. Each trapping grid was 9 ha in size with 50 traps arranged on 10 transects (5 traps/transect) in a rectangular pattern with 50 m between traps. The two trapping grids were separated by 1.5 km of poor hare habitat (e.g., open muskeg, wetland). No hares were observed moving between grids during 12 years of population monitoring. However, it was not uncommon for study hares to move up to 1 km from the trapping grids and we observed 5 hares moving more than 5 km from their respective capture sites [16].

## Methods

### Snowshoe Hare Capture and Collaring

We captured hares in #3 Havahart live traps (model 1085, Lititz, PA) and marked them with Monel ear tags (National Band and Tag Co., Newport, KY) for use in ongoing population estimates. Traps were baited with alfalfa and carrots, provisioned with snow (when available) for moisture, and were opened during mid-day and checked the following morning. Trapping did not take place at temperatures below -18°C. Capture and handling of snowshoe hares followed animal care and use guidelines of the American Society of Mammalogists [33] and were approved by the University of Alaska Fairbanks Institutional Animal Care and Use Committee (protocol #09–57) and the Alaska Department of Fish and Game (Permit 135211–5).

Between May 2008 and August 2012, we radio-tagged with VHF transmitters a subset of the hares captured during routine 4-night trapping sessions in June and September for population estimates, and during 1- to 3-night trapping sessions conducted as needed in all seasons to deploy radio transmitters when sample size was reduced by attrition. We initially fitted 8 and 12 hares in Deciduous and Conifer, respectively, with VHF radio transmitters in June 2008 and increased the sample to 20 individuals per grid by September. Transmitters weighed 20–26 g (models M1555, M1565, M1575, Advanced Telemetry Systems, Isanti, MN) and were equipped with a mortality switch activated by a lack of movement after 6 consecutive hours. Collars were only put on hares weighing > 900 g so that they did not exceed 3% of the hare’s body weight; this restricted our study to adult and older juvenile hares.

**Fig 2:**
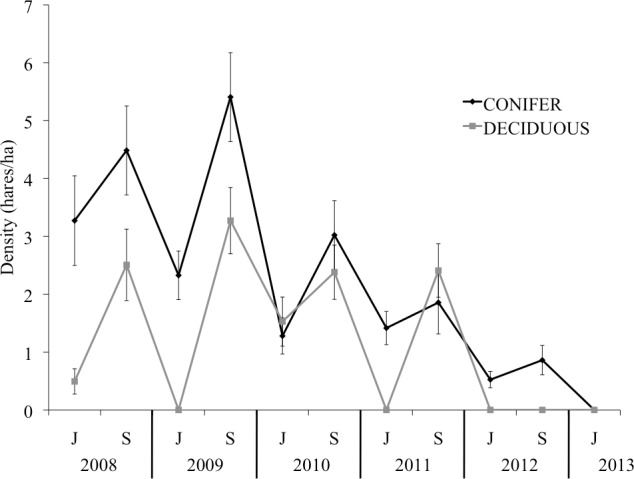
Estimated snowshoe hare densities in Bonanza Creek Experimental Forest near Fairbanks, Alaska, from June 2008 to June 2013. Estimates were based on live-capture of ear-tagged hares in June (J) and September (S) of each year. Error bars show SE.

We redeployed collars on new hares as mortalities occurred in an attempt to maintain at least 25 collared individuals in each site at any given time. Collared hares represented 20–90% of the estimated hare population on each trap grid, depending on the time of year. On the basis of an ongoing mark-recapture study, hare densities peaked at 5.4 and 3.3 hares/ha in the Conifer and Deciduous sites, respectively, in autumn 2009 (Fig 2). Densities fell to approximately 2 hares/ha on both trap sites in autumn 2011, and by spring of 2012 were < 1 hare/ha. Hares were collared through June 2012 in Conifer; however, hare abundance was too low in Deciduous to collar additional hares after November 2011. Fewer than 5 collared hares remained in Deciduous by mid-December 2011 and none by mid-May 2012.

Up to 6 hares were fitted with collars equipped with GPS loggers (model G30L, Advanced Telemetry Systems; model Quantum 4000, Telemetry Solutions, Concord, CA) and VHF transmitters in Deciduous between June and October of 2010 and May and September 2011, and in Conifer between February and April 2012, for a concurrent study on hare movement and activity patterns [16]. GPS collars were removed and replaced with VHF collars when GPS batteries expired, usually after 2–3 weeks. The maximum weight of GPS collars was 40 g (4% of a hare’s mass) and not expected to affect survival [33], so we included data from individuals fitted with GPS collars in our survival estimates.

### Monitoring and Mortality Identification

We monitored VHF-collared hares (including those with GPS) every 1–7 days using a directional Yagi antenna and hand-held receiver (model R1000 receiver; Communications Specialists Inc., Orange, California) to detect movement of hares off the grids and locate mortalities. When transmitter signal strength or location suggested that a hare had moved > 500 m from its trapping grid, we attempted to find and visually confirm its location. We right-censored (i.e. removed from the study) hares with transmitters that stopped functioning.

Mortalities were typically located within 1 week of death and their locations were recorded using a handheld GPS unit. Sources of predation were identified whenever possible using methods by Krebs et al. [24]. Lynx (*Lynx canadensis*) and coyote (*Canis latrans*) predation was primarily identified by tracks or the method of caching a carcass. Northern goshawk (*Accipiter gentilis*) predation was identified by the presence of long, thin mutes at the kill site, plucked fur and tendons, and intact skeletal remains. Great horned owl (*Bubo virginianus*) predation was identified by rounded, cream colored mutes, and decapitation. We also used field sign such as hair, feathers, scat, wing marks, regurgitated pellets, and portions of the hare consumed to distinguish among kills made by these primary predators and other species. Because field sign left by scavengers could be misidentified as sign of predation, we monitored hare carcasses in the trapping grids using trail cameras. Carcasses were generally not scavenged until 10 or more days following death, which is similar to patterns observed in the Yukon Territory [34]. Additionally, the majority of scavenging was done by animals typically incapable of preying on older juvenile or adult hares, such as common raven (*Corvus corax*), gray jay (*Perisoreus canadensis*) or red squirrel (*Tamiasciurus hudsonicus*). Lynx and northern goshawks were occasionally observed scavenging carcasses, but only after other species had already consumed most of the carcass. Deceased hares lacking external signs of predation were necropsied for signs of subcutaneous bruising or puncturing that would indicate predation. If none were found, we considered the cause of death to be non-predatory.

### Structural and Vegetative Cover

In order to evaluate relationships between structural cover and hare survival, we measured horizontal cover (i.e. visual obstruction) and canopy cover at 50 locations in each trapping grid: in spring when both snow and deciduous leaves were absent (May); summer (July-August); and winter, when snow depth was greatest (March-April). We assumed structural cover to be similar in fall and spring because neither deciduous leaves nor snow were present at these times. Five sampling points were selected at random distances along each of the 10 established transect lines in the trapping grids. We also measured canopy cover and horizontal cover at each hare predation site in the season the predation event occurred if we were confident the carcass remains had not been moved by predators or scavengers.

We measured canopy cover at each sampling point and predation site using a concave spherical densiometer [35]. Horizontal cover was measured as the percentage of a 0.3 x 2.5 m profile board obscured by vegetation at distances of 5, 10, 15, 20, 25, and 30 m. Preliminary analysis indicated that horizontal cover was most variable among habitats at a distance of 10 m, so we used data from only this distance in later analysis. At predation sites, we averaged horizontal cover measured in the 4 cardinal directions. At sampling points in the grids, we randomly selected a direction perpendicular to the transect line and conducted measurements directly adjacent to the transect where vegetation had not been impacted by foot traffic. We recorded horizontal cover from ground level (or top of the snowpack in winter) to a height of 2.5 m in order to account for vegetative obstruction to both terrestrial and avian predators [13, 15]. Measurements were taken in five 0.5-m high sections [36], where each section was subdivided into 4 quadrants to improve reading accuracy, then averaged across the entire 2.5 m. We observed the board from 0–1.0 m above ground from a kneeling position, and from 1.0–2.5 m from a standing position, in order to maintain a nearly horizontal viewing plane.

In addition to measuring horizontal cover and canopy cover in the trapping grids, we did so in a stand of mature mixed white spruce-birch forest (hereafter referred to as “Mixed”) located adjacent to Conifer after we observed frequent use of this stand by study hares collared in Conifer. We established a 14 ha grid with 50 m between sampling points and took measurements in summer and winter only, assuming similar values for spring, fall, and winter, based on trends in the other sampling grids.

### Analyses

We investigated the importance of biotic and abiotic variables to snowshoe hare survival from 10 June 2008 to 31 May 2012 using known-fate models with a daily interval in program MARK [37]. This allowed for staggered entry of new animals and censoring of individuals whose transmitters failed [38, 39]. Data from censored individuals were used in the models up until the time of censoring. We examined the effects of time, capture site, age, sex, body condition, and environmental variables (Table 1) on daily survival rates. We included variables for month, season, and year in separate models to address the importance of temporal fluctuations in survival at different scales. Due to major differences in vegetation (and therefore cover and food availability) among trapping grids, we allowed for different survival rates between capture sites. We also allowed for differences in survival between juvenile and adult hares, and males and females, due to behavioral differences such as movement rates and lack of vigilance that could lead to different rates of predation. We included a morphometric variable (mass/hind foot length) as a measure of body condition with the expectation that a hare’s mass, after adjusting for skeletal size, is directly related to its ability to escape predation and survive extreme weather. Mass and hind foot length are strongly correlated with body condition in hares and these measures are robust to differences in sex and age [40]. We found no relationship between this body condition index and skeletal size within a given age and sex of hare, indicating that the index was unbiased (S1 Table). Finally, we included variables for air temperature, the presence and depth of snow, and precipitation falling as snow, because we expected these weather parameters to affect a hare’s decision and ability to forage, maintain homeothermy, and escape predation (S2 Table). We formed a set of 85 models that included additive models with up to 3 variables or simple interaction models containing only 2 variables (S3 Table). While this may be considered an exploratory analysis due to the relatively large number of models that were used to evaluate multiple hypotheses, we constructed the model set before the data were analyzed and otherwise treated the analysis as *a priori*, ensuring that sample size was sufficient for each model, correlated variables did not appear together in any model, and each model was biologically justifiable based on previous research and knowledge of the biological system. We compared models using Akaike’s information criterion corrected for small sample size (AIC_c_) and Akaike model weights [41]. Goodness-of-fit testing is not available for known-fate data with individual covariates [42], so we assumed little to no overdispersion in the data and used a value of 1.0 for the overdispersion parameter c-hat. However, we compared model rankings obtained using c-hat values of 1.0, 2.0, and 3.0 to gauge the potential effects of any unexpected overdispersion.

**Table 1:** Variables used to construct known-fate survival models for snowshoe hares. Hares were collared in Bonanza Creek Experimental Forest near Fairbanks, Alaska, from June 2008 to May 2012.

Parameter	Description
Age	Age (juvenile or adult) at time of radio-tagging. Juveniles became adults after March 1. Juveniles were distinguished from adults through mid-September using a combination of hind foot length, mass, and pelage color. After mid-September, hares of unknown age were categorized as adults.
Sex	Sex (male, female, or unknown).
Body condition	An index of body condition calculated as weight divided by hind foot length. If a hare was captured more than once, measurements for weight and hind foot were averaged over the time that hare carried a radio transmitter.
Site	Trapping grid (Deciduous or Conifer) in which hare was radio-tagged.
Month	Calendar month.
Season	Summer (~1 Jun to ~1 Sep) was defined by the presence of deciduous leaves and absence of snow. Fall (~1 Sep to ~15 Oct) was defined by the senescence of deciduous leaves and absence of snow. Winter (~15 Oct to ~1 May) was defined by the absence of deciduous leaves and presence of snow. Spring (~1 May to ~1 Jun) was defined by the absence of both deciduous leaves and snow.Start and end dates varied slightly each year based on the presence of deciduous leaves and snow.
Year	Annual period from 1 June to 31 May, coinciding with the approximate parturition of first litters.
Air temperature*	Average air temp at 50 cm above ground when snow depth is <50 cm, or 150 cm above ground when snow depth is >50 cm.
Snow presence*	Presence/absence of at least 0.5 cm of snow on ground persisting for more than 1 day. Once present, snow cover was continuous through winter, making this a binary variable for season.
Snow depth*	Average depth of snow on ground during monitoring interval, measured to 0.1 cm.
Snowfall*	Total precipitation falling as snow during monitoring interval, measured to 0.1 cm.

*Weather data were collected by Bonanza Creek LTER at a weather station located 500 m from the Deciduous trapping grid and 1.5 km from the Conifer grid. These data are summarized in S2 Table.

We tested for differences in the number of avian and mammalian predation events among the predominant habitats in which hares died (black spruce, early successional, and mixed forest) by comparing the number of hares killed by each predator class in each habitat using a Chi-Square test of independence. We also report the number of predation events by predator species and those occurring in other habitats, but we did not conduct Chi-Square tests using these habitats or predator designations due to insufficient sample size.

To test for seasonal changes in vegetative cover in hare habitats, we compared canopy cover and horizontal cover among the Conifer, Deciduous, and Mixed grids using repeated measures analysis of variance with the Greenhouse-Geisser adjusted F-test to account for a violation of sphericity, followed by Tukey’s HSD multiple comparisons. Canopy cover and horizontal cover measurements were arcsine square root transformed before analysis. We report summary statistics for canopy cover and horizontal cover at predation sites, but statistical comparisons to random samples were not conducted due to incompatible sampling designs. The vegetation communities in the Conifer, Deciduous, and Mixed grids were similar in structure and seral stage to other black spruce, early successional, and mature mixed forests, respectively, in the study area.

Unless otherwise indicated, we used the statistical program JMP (Version 7. SAS Institute Inc., Cary, NC, 1989–2007) for analyses, used an alpha of 0.05 in assigning statistical significance, and report means with standard error.

## Results

### Snowshoe Hare Survival

We radio-tagged a total of 288 hares between 10 June 2008 and 31 May 2012 (Table 2). The support for the most parsimonious survival model, *S* (site + body condition + month), was 52.9%, and it was 4.5 times more likely to be the best model than the model with the next highest AIC_c_ weight (Table 3). The top model indicated that (1) survival rate differed among months in the year, (2) hares radio-tagged in Conifer were more likely to survive than hares tagged in Deciduous, and (3) hares with a higher body condition index had higher survival rates. Month and site were components of all models with Δ AIC_c_ values < 4.0, and had summed model weights of 0.862 and 0.841, respectively. Body condition was a variable in 3 of 7 models with Δ AIC_c_ values < 7.0 and had a summed model weight of 0.661. Month, site, and body condition explained most of the variation in hare survival in our study (Table 4). There was less support for higher survival of adults than juveniles. However, this is a conservative estimate of the importance of age to hare survival because classifying hares of unknown age as adults (as we did) will tend to obscure any real difference that may exist between age classes. There was some support for differences in survival among seasons, indicating a possibility of higher survival of hares in summer and fall than in winter and spring. We found very little support for a difference in survival between sexes, among study years, or in relation to measures of snow cover or air temperature.

**Table 2:** Classification and fates of radio-tagged snowshoe hares. Hares were collared in the Conifer and Deciduous trapping grids in Bonanza Creek Experimental Forest near Fairbanks, Alaska, from June 2008 to May 2012.

	Conifer	Deciduous
**Total collared**	159	129
**Male/female/unknown**	76/80/3	37/88/4
**Adult/juvenile/unknown**	92/12/55	83/15/31
**Fate**		
** Predation mortality**	84	66
** Starvation mortality**	4	4
** Unknown mortality**	16	32
** Censored (e.g. lost transmitter signal)**	36	27
** Alive when the study ended**	19	0

**Table 3:** The 15 highest ranked known-fate models of snowshoe hare survival. Hares were collared in Bonanza Creek Experimental Forest near Fairbanks, Alaska, from June 2008 to May 2012.

Model	AIC_c_ ^a^	Δ AIC_c_	AIC_c_ weight	Model likelihood	K^b^
S (site + month + body condition)	1720.35	0.00	0.529	1.000	14
S (site + month + age)	1723.36	3.01	0.117	0.222	14
S (site + month)	1723.95	3.60	0.088	0.166	13
S (site + body condition + season)	1724.25	4.07	0.069	0.131	6
S (month + body condition)	1724.78	4.43	0.058	0.109	13
S (month + age)	1725.42	5.06	0.042	0.080	13
S (month)	1727.10	6.75	0.018	0.034	12
S (site + season)	1727.94	7.59	0.012	0.023	5
S (site * season)	1727.97	7.62	0.012	0.022	8
S (site + season + age)	1728.26	7.91	0.010	0.019	6
S (body condition + season)	1728.83	8.47	0.008	0.015	5
S (month * age)	1729.56	9.20	0.005	0.010	23
S (month + sex)	1730.25	9.90	0.004	0.007	14
S (season + age)	1730.59	10.24	0.003	0.006	5
S (season)	1731.06	10.70	0.003	0.005	4

^a^Akaike’s Information Criterion adjusted for small sample size.

^b^Number of parameters.

**Table 4:** Summed model weights for variables in known-fate models of snowshoe hare survival. Hares were collared in Bonanza Creek Experimental Forest near Fairbanks, Alaska, from June 2008 to May 2012. Model weights are summed over all 85 models in the model set.

Variable	Weight	# Models
Month	0.862	10
Site	0.841	36
Body condition	0.667	20
Age	0.181	23
Season	0.119	10
Sex	0.004	13
Air temperature	0.001	22
Snow presence	0.003	12
Snow depth	0	12
Snowfall	0	12
Year	0	10

**Fig 3:**
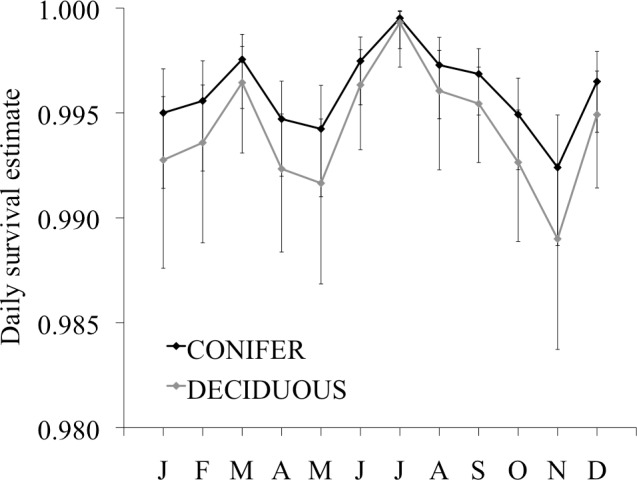
Daily survival rate estimates for snowshoe hares. Hares were collared in the Conifer and Deciduous trapping grids in Bonanza Creek Experimental Forest near Fairbanks, Alaska, from June 2008 to May 2012. Estimates are based on the model *S* (Body Condition + Site + Month) and are reported for a mean body condition index of 10.4. Error bars represent 95% *CI*.

**Fig 4:**
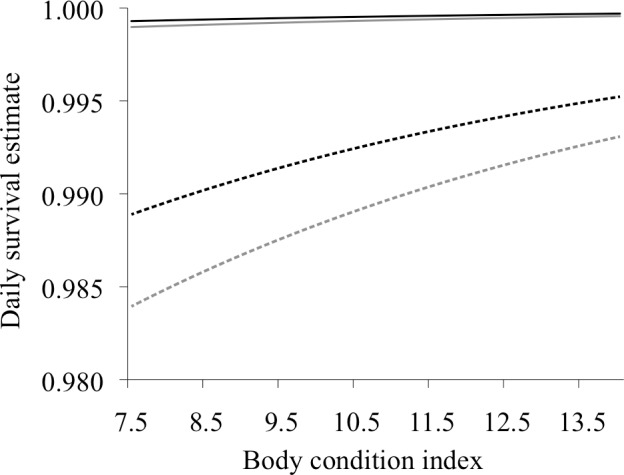
Relationship between estimated daily survival rate and body condition index for snowshoe hares. Estimates are shown for hares collared in the Conifer (black lines) and Deciduous (grey lines) trapping grids in July (solid lines) and November (dashed lines) (the months of highest and lowest snowshoe hare survival) in Bonanza Creek Experimental Forest near Fairbanks, Alaska, from June 2008 to May 2012. Estimates are based on the model *S* (Body Condition + Site + Month). Confidence intervals (95%, not shown) indicated some overlap between trapping grids within a season.

Estimated daily survival rate (based on an average body condition index of 10.4) was highest in July for hares tagged in both trapping grids (Conifer: 0.9995 ± 0.0003; Deciduous: 0.9993 ± 0.0005) and lowest in November (Conifer: 0.9924 ± 0.0015; Deciduous: 0.9890 ± 0.0022; Fig 3). These values equate to an estimated 30-day survival rate of ~0.98 in July for hares in both trapping grids, and 0.80 and 0.72 in November for Conifer and Deciduous, respectively. Survival differed most between trapping grids in November when estimated survival was lowest, but there was a high level of variability in monthly estimates and considerable overlap between sites (Fig 3). Body condition had a smaller effect on estimated daily survival rates during months where survival was high, such that body condition made little difference to daily survival in July and had the greatest influence on survival in May and November (Fig 4). Because hind foot length did not differ greatly between sexes or age groups, adult females averaged a 10% higher body condition index than adult males due to additional weight, and adults averaged a 13% higher body condition than juveniles for the same reason (S1 Table).

Despite the lack of model support for differences in survival among years, we note that annual survival based on product limit estimation increased from 0.12 ± 0.04 in 2008–09 to 0.29 ± 0.03 in 2009–10 and 0.24 ± 0.08 in 2010–11, before declining to 0.08 ± 0.08 in 2011–12. Estimated annual survival was 5–17% higher for hares tagged in Conifer than in Deciduous in all years.

When adjusting the overdispersion parameter c-hat to a value of 2.0 or 3.0, the model rankings still reflected the importance of a temporal component, but emphasized seasonal differences in survival rather than monthly differences. Site was still included in many of the top models, and body condition and age remained important individual covariates. We conclude that using a c-hat = 1.0 was appropriate for the data.

### Sources of Predation

**Fig 5:**
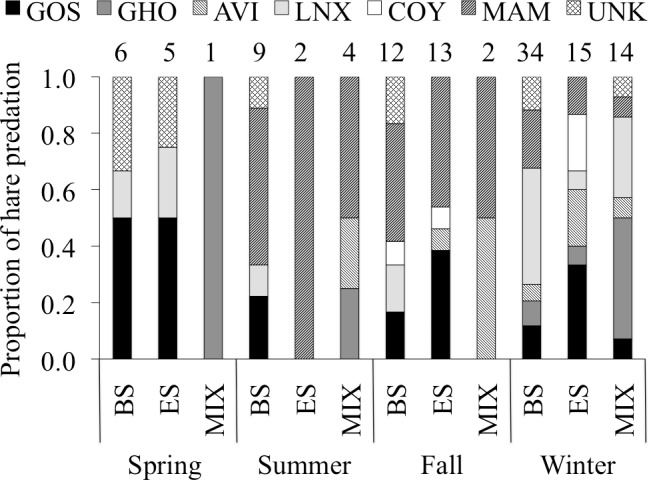
Proportion of snowshoe hare predation by predator species, season, and habitat type. Hares were collared in Bonanza Creek Experimental Forest near Fairbanks, Alaska, from June 2008 to May 2012. Habitat types are black spruce forest (BS), early successional forest (ES), and mixed forest (MIX). Only the habitats most frequently used by hares in our study are shown. Predators are goshawk (GOS), great horned owl (GHO), unidentified raptor (AVI), lynx (LNX), coyote (COY), and unidentified mammal (MAM). Sample size is given above each column.

We documented 149 predation events, 129 of which we could identify as mammalian or avian predation, and 85 of which we could identify the predator species (Fig 5). We observed slightly more predation of hares by mammals than by avian predators. Lynx, goshawks, and great horned owls were responsible for the majority of kills for which we could identify the predator species, whereas coyote predation was rarely observed. There was little evidence of predation by any other species present in the study area (e.g., red fox (*Vulpes vulpes*), mink (*Mustela vison*), ermine (*M*. *erminea*), and marten (*Martes americana*). In 2 of 3 cases where mink or ermine were associated with hare mortalities, the carcasses were cached intact and were likely scavenged by the weasels. We could not identify the predator species for 43% of hare kills, typically when hares had been killed by mammals in weather conditions that did not allow for track identification (e.g., in the absence of snow, after tracks had been obscured by heavy snowfall, or after tracks had melted in warm spring conditions).

Hares were killed almost twice as often in black spruce forests as in early successional forests and three times more frequently in black spruce forests than in mixed forests (Table 5). There was no difference in the proportion of hare predation by mammalian and avian predators among black spruce, early successional, or mixed forests (χ^2^ = 0.455, df = 2, *P* = 0.103).

**Table 5:** Number of predation events by habitat type for snowshoe hares. Percents given in parentheses represent the amount of predation observed in a given habitat for the predator listed at the top of the column. Hares were collared in Bonanza Creek Experimental Forest near Fairbanks, Alaska, from June 2008 to May 2012.

Habitat	Predation	Avian	Mammalian	Goshawk	Great Horned Owl	Lynx	Coyote
Black Spruce	61 (41%)	16 (28%)	36 (51%)	11 (38%)	3 (16%)	18 (60%)	1 (14%)
Early Successional	34 (23%)	17 (29%)	16 (23%)	12 (41%)	1 (5%)	2 (7%)	4 (57%)
Mixed Forest	21 (14%)	12 (21%)	8 (11%)	1 (3%)	8 (42%)	4 (13%)	0 (0%)
White Spruce	13 (9%)	6 (10%)	3 (4%)	4 (14%)	1 (5%)	2 (7%)	1 (14%)
Young Birch	11 (7%)	4 (7%)	4 (6%)	1 (3%)	3 (16%)	3 (10%)	0 (0%)
Shrub	8 (5%)	3 (5%)	3 (4%)	0 (0%)	3 (16%)	1 (3%)	0 (0%)
Ice	1 (1%)	0 (0%)	1 (1%)	0 (0%)	0 (0%)	0 (0%)	1 (14%)

However, we documented over twice as many kills by mammalian predators than by avian predators in black spruce forests, likely resulting from lynx predation, which was >4 times more frequent in black spruce forests than in any other habitat. We note that study hares on the trapping grids had more immediate access to black spruce forests than other habitat types (Fig 1). Of the predator-killed hares that were radio-tagged in Conifer (black spruce forest), 61% died in black spruce forests, 23% in mixed forests, and none in early successional forests. Only 40% of predator-killed hares radio-tagged in Deciduous (early successional forest) died in early successional forests, whereas 27% died in black spruce forests.

The majority of hare predation (56%) took place during winter (Fig 5), but after accounting for different season lengths, predation rates (number per month) were roughly equal in spring, fall, and winter, and very low during the summer. Avian predation rates were highest in spring, owing to a large amount of goshawk predation. However, great horned owl predation rates were highest in winter. Mammalian predation rates were highest in fall and winter, and very low in spring and summer, but tracks were not available for identification in the latter seasons. The number of hares killed by lynx in winter was more than double that in any other season, and coyote predation was only identified in fall and winter. We observed ~10–25% more predation of hares by mammals than birds in all seasons except spring, when avian predation was 4 times more frequent than mammalian predation.

The percent of hares killed by avian predators in the study area rose from 28% in 2008–09 to 49% in 2011–12, while mammalian predation fell from 56% to 41% over the same time period. The percent of predation from goshawks was relatively consistent across study years, whereas predation from great horned owls rose from 6% in 2008–09 to 27% in 2011–12. Lynx predation declined from 33% to 7% during the same time period, whereas coyote predation was at its peak (12%) in 2011–12.

### Structural Cover

There was more canopy cover in summer than in other seasons in all sampling grids, but the Deciduous and Mixed grids exhibited the greatest seasonal changes (Table 6). During summer, canopy cover in Mixed = Deciduous > Conifer, and in winter Mixed > Conifer > Deciduous. Horizontal cover was considerably greater in all sampling grids in summer than in other seasons and Conifer > Deciduous > Mixed in all seasons (Table 6).

**Table 6:** Mean ± SE for percent canopy cover and horizontal cover in primary snowshoe hare habitats. Sampling grids were located in Bonanza Creek Experimental Forest near Fairbanks, Alaska. Cover was measured between June 2008 and May 2012.

	Conifer	Deciduous	Mixed
**Canopy cover**			
** Winter**	26.4 ± 2.5^a^	13.2 ± 1.3^c^	46.9 ± 1.8^e^
** Spring**	26.7 ± 2.1^a,b^	14.6 ± 1.0^c^	
** Summer**	35.0 ± 2.7^b^	73.9 ± 2.9^d^	75.9 ± 1.2^d^
**Horizontal cover**			
** Winter**	66.5 ± 2.9^f^	27.9 ± 3.3^i^	8.9 ± 1.3^k^
** Spring**	57.6 ± 3.7^g^	21.0 ± 2.3^i^	
** Summer**	89.8 ± 2.4^h^	71.0 ± 3.5^j^	28.5 ± 2.4^l^

Letters indicate groups that differ at alpha = 0.05 for seasons compared within a site, and for sites compared within a season.

We measured structural cover in 122 predation sites, 50 of which were in black spruce forests, 33 in early successional forests, 17 in mixed forests, and the remainder in white spruce, birch, shrub, wetland, or on snow-covered ice. Due to our sampling design, we did not conduct statistical analyses comparing cover at predation sites to available cover. We note that hare predation within the bounds of the trapping grids during fall took place in locations averaging 34% and 15% less horizontal cover than the random samples in Conifer (7 kill sites) and Deciduous (13 kill sites), respectively. This trend also extended to black spruce and early successional forests in the study area (Table 7). Horizontal cover was on average 32% greater at predation sites than random samples in Mixed (9 kill sites), but otherwise did not differ from random samples. Canopy cover at predation sites (Table 8) did not differ from random samples.

**Table 7:** Mean ± SE for percent horizontal cover at snowshoe hare predation sites in primary hare habitats. Predation sites were located in Bonanza Creek Experimental Forest near Fairbanks, Alaska, between June 2008 and May 2012. Sample sizes are shown in parentheses.

	Black Spruce					Early Successional					Mixed Forest			
	Spring	Summer	Fall	Winter		Spring	Summer	Fall	Winter		Spring	Summer	Fall	Winter
Predation	55 ± 10 (6)	18 ± 4 (6)	10 ± 3 (8)	40 ± 5 (30)		57 ± 15 (3)	16 (1)	58 ± 5 (16)	66 ± 7 (13)		78 (1)	67 ± 6 (4)	53 ± 20 (3)	79 ± 3 (9)
Avian	76 ± 2 (2)		0 (1)	41 ± 6 (12)		45 ± 15 (2)		56 ± 7 (7)	67 ± 10 (7)		78 (1)	73 ± 6 (2)	37 ± 21 (2)	77 ± 5 (5)
Mammalian	48 ± 21 (2)	21 ± 6 (4)	12 ± 3 (7)	33 ± 7 (14)		82 (1)	16 (1)	62 ± 8 (8)	78 ± 22 (2)			62 ± 11 (2)	85 (1)	82 ± 3 (2)
Goshawk	76 ± 2 (2)		0 (1)	44 ± 7 (6)		45 ± 15 (2)		54 ± 7 (6)	64 ± 11 (6)				15 (1)	75 (1)
Great Horned Owl				47 ± 19 (3)					85 (1)		78 (1)			79 ± 9 (3)
Lynx	69 (1)	14 (1)	6 (1)	36 ± 8 (9)		82 (1)		75 (1)						82 ± 3 (2)

**Table 8:** Mean ± SE for percent canopy cover at snowshoe hare predation sites in primary hare habitats. Predation sites were located in Bonanza Creek Experimental Forest near Fairbanks, Alaska, between June 2008 and May 2012. Sample sizes are shown in parentheses.

	Black Spruce					Early Successional					Mixed Forest			
	Spring	Summer	Fall	Winter		Spring	Summer	Fall	Winter		Spring	Summer	Fall	Winter
Predation	49 ± 9 (7)	56 ± 5 (5)	29 ± 6 (8)	30 ± 4 (29)		54 ± 37 (2)	52 (1)	19 ± 4 (16)	12 ± 3 (14)		93 (1)	77 ± 5 (5)	59 ± 19 (3)	61 ± 6 (10)
Avian	29 ± 27 (2)		17 (1)	27 ± 4 (11)		90 (1)		27 ± 6 (7)	12 ± 2 (8)		93 (1)	76 ± 5 (3)	48 ± 26 (2)	66 ± 5 (6)
Mammalian	60 ± 4 (3)	60 ± 9 (3)	31 ± 7 (7)	37 ± 8 (14)		17 (1)	52 (1)	11 ± 2 (8)	3 ± 3 (2)			79 ± 12 (2)	82 (1)	60 ± 26 (2)
Goshawk	29 ± 27 (2)		17 (1)	35 ± 5 (5)		90 (1)		29 ± 7 (6)	11 ± 2 (7)				22 (1)	77 ± 1 (2)
Great Horned Owl				15 ± 8 (3)					13 (1)		93 (1)	85 (1)		56 ± 7 (3)
Lynx	55 (1)	45 (1)	38 (1)	32 ± 9 (9)		17 (1)		16 (1)						60 ± 26 (2)

## Discussion

### Survival Models

Estimated daily survival rates of snowshoe hares were higher in summer than in other seasons, which was likely associated with greater vegetative cover across all habitats and lower predation rates [34]. From May through August each year, there presumably were leverets and newly weaned juvenile hares in the area, which would have served as a more accessible food source for predators that might have otherwise captured older hares [43]. We speculate that this change in prey availability temporarily alleviated the rate of predation on the older age classes for which we estimated survival rates. Alternative prey sources such as small mammals and migratory birds are also in greater abundance during the summer, which may have further contributed to the lower predation rates on hares [44–46].

Depressed survival rates in April and May coincide with the vernal pelage change and an increase in activity associated with breeding behavior, such as movement of males to find mates [47, 48]. In the absence of deciduous cover and with poor camouflage during snowmelt [49], hares are more visible to predators during this early spring period. Declining survival rates of hares in October and November may reflect a similar scenario where autumnal molting and sparse cover make hares more visible to predators. There is evidence that hares with white or mottled pelage use areas with denser vegetative cover than hares with brown fur during times when snow is not present [50]. Thus, hares may shift habitat use toward dense conifer forest in response to increased vulnerability to predation during molting periods, although predation continues to take place in such dense vegetation.

Differences in age-specific survival rates may also contribute to lower overall estimated survival rates in fall. Beginning in September each year, we introduced juveniles to our sample of radio-collared hares after they reached weights of 900 g. If juveniles have lower survival rates than adults, as was shown in other studies [8, 51–53] and for which we have evidence here and previously [32], then we would expect a drop in estimated survival rates when younger juveniles are added to a sample population comprised entirely of adult hares.

Fall also represents a time of physiological transition and stress for hares as their diet shifts from fresh foliage to one comprised mostly of woody browse, mean daily air temperature drops below freezing, and snow begins to accumulate. All of these factors are likely to reduce survival via changes in behavior and physiology. By comparison, hares experiencing cold winter temperatures (-20°C) in western Canada exhibited lower resting and field metabolic rates, thermal conductance, and lower critical temperatures than hares in fall [23, 54], suggesting that hares are better adapted to winter conditions. The energetic demands of molting into denser and longer winter pelage [54] while remaining vulnerable to environmental stressors in fall may temporarily lead to depressed body condition and ultimately to either starvation [31] or predation [7].

Our models (Table 2) indicated higher survival of hares that were radio-tagged in a black spruce forest (Conifer) than hares tagged in an early successional forest (Deciduous), with the greatest difference occurring during times when deciduous leaves were absent. With the vast majority of hare mortalities resulting from predation, the structural cover available to hares in black spruce forest likely offered considerably more protection from predators than the open habitat of the early successional forest. However, over half of the hares that died were found beyond the boundaries of the trapping grids, often in markedly different habitat than where the hares had been radio-tagged. Due to logistic constraints, we were unable to regularly locate all individuals that moved beyond the grid boundaries, and therefore can only attribute survival rates to the grid in which a hare was initially radio-tagged. Bihourly relocation data from GPS-collared hares in our study indicated that hares regularly moved among habitats (i.e. away from the trapping grids), often on a daily basis, and presumably to forage or seek refuge [16]. Hares in Montana also made regular movements between dense and open vegetation types, exhibiting higher survival rates in dense mature forest types [8]. Moving between adjacent habitats or using habitat edges is one solution to optimizing both browse quality and the availability of escape cover. For this reason, the home range of any individual hare in a landscape with small patch sizes, such as our study area (Fig 1), is likely to span multiple habitats [16]. In our study area, mature black spruce forest likely represented the densest stand type consistently inhabited by hares, whereas early successional forest offered close to the minimum cover necessary for hare occupancy. The hare densities and survival rates in our study suggest that mature black spruce forest serves as a refuge during peak predator densities and in seasons when deciduous cover is absent. In terms of survival, the protection conferred by vegetative cover in black spruce forest appears to outweigh any cost of browse availability, especially when alternative foraging opportunities exist nearby. This is in agreement with previous findings that showed lower predation rates for hares in habitats with less visibility [15].

There was no support for differences in survival among study years in our models despite a 10-fold variation in hare density during the study and large changes in annual survival rates observed in other studies (0.5–32%) [55]. However, our models compared daily survival estimates among years rather than cumulative annual survival estimates, which may explain the relatively low level of support in the model set. Survival rates were highest around the population peak, as they have been elsewhere [17–19], but they decreased less than we had expected based on observations in Kluane [55]. Predator-induced stress in hares, which peaks with predator abundance, can lead to lower birth rates and fewer viable young for females in the decline phase of the population cycle [23, 56, 57]. Given that non-predatory sources of mortality represented a minor proportion of hare deaths throughout our study, as has been found elsewhere [55], we surmise that the sharp population decline was due to reduced recruitment attributable to either lower fecundity, lower leveret/juvenile survival, or a combination thereof.

Our survival models suggest that higher body condition in hares was associated with higher survival rates, especially during months of low survival and for hares from the Deciduous grid (early successional forest). Given that 95% of hare deaths for which the cause was known were due to predation, higher body mass for a given skeletal size (most likely resulting from greater muscle mass) probably conferred an advantage for avoiding predation. This might come about through diminished foraging times and minimized exposure to predators, or by physical ability to escape predators in a chase situation. Hares with access to high quality browse should be more likely to maintain their body weight throughout the winter and spend less time foraging to do so. The apparent winter diet (based on fecal pellet analysis) of hares in the Conifer grid was dominated by spruce with a minor component of birch [58]. By contrast, hares in the Deciduous grid had a more diversified diet comprised of willow, balsam poplar, alder, and spruce, indicating greater availability of high-quality winter forage in and around this early successional habitat. In spite of these differences, average body condition was similar for hares in Conifer and Deciduous. Thus, we suggest that body condition affected hares from Deciduous more than those from Conifer because hares from Deciduous encountered more predation scenarios in seasons when deciduous leaves were not available for cover. Here, we need to reiterate that the body condition index value for hares captured on multiple occasions was based on an average weight across those occasions. Hares tend to lose mass through the winter [32, 59], so allowing weight to change monthly for individuals in our survival models would have been more informative than using a single, average weight for estimating the importance of body condition to hare survival. However, recapture rates for collared hares were inconsistent and weights were often taken months apart for individuals. Rather than using only the last weight taken before a hare died, which was often recorded in a different season than when mortality occurred, we averaged available weights, which changed approximately 12% between seasonal trapping sessions. By comparison, hares averaged 5% changes in weight between consecutive trapping nights, due largely to bait consumption or bowel evacuation. We did not palpate or otherwise attempt to identify pregnancy in females, so it is possible that weights from pregnant females were used in calculations of their average body weights, which would have resulted in assignment of a slightly higher body condition. However, because we do next expect pregnancy to confer a survival advantage, any effect of including the weights of pregnant females should diminish the relative importance of body condition as an explanatory variable in our model, making our estimates of this parameter conservative.

### Sources and Sites of Predation

Snowshoe hares are the predominant prey species for lynx, coyotes, northern goshawks, and great horned owls [55, 60, 61], and predation by these species accounted for the majority of hare mortality in our study area. However, we observed less coyote predation than expected based on findings in central and western Canada [2, 45, 46, 62] in spite of a fairly common occurrence of coyote scat and tracks in and around our trapping sites throughout the year. We could not identify the predator species in nearly half of the hare deaths by mammalian predators, but we have no reason to believe that the proportion of kills by each predator would differ from those that were positively identified. Evidence of mammalian predation (carcass remains) was essentially the same as for lynx and coyote predation, but lacked tracks for species identification.

We documented seasonal differences in sources of hare predation that mirrored observations made elsewhere [21, 22, 61]. Hare predation by coyotes was limited to fall and winter, and predation by lynx, goshawks, and great horned owls largely took place during winter and spring. We also observed changes in predation during the peak and decline phase of the hare cycle, which might be attributed to numeric and/or functional responses of predators in relation to hare densities [19–21]. Without direct estimates of predator abundance in the area, it is difficult to say whether the changes in predation were due to local movement of individual predators or to a more widespread trend in populations. Trapping pressure on lynx was intense in our study area and might explain the decrease in lynx predation by way of a reduction in local abundance of these predators. In an ongoing companion study of lynx in the Bonanza Creek study area, all deaths of collared lynx (n = 22) were due to trapping during the same study period (K. Kielland, University of Alaska Fairbanks, unpublished data). This might also have allowed coyotes to make greater use of areas otherwise hunted by lynx, as has been hypothesized in another study [11]. Moreover, fur sealing data from the Alaska Department of Fish and Game indicate that lynx harvest declined 64% from 2009–10 to 2011–12 in the game management unit containing our study area [63], which suggests a declining lynx population. With the vast majority of lynx being trapped between December and February [63], hares may have experienced dramatically lower predation pressure in winter, leading to the unexpectedly high survival rates we observed during this season. Coyotes were considered by trappers to be scarce during the winter and their harvest was very low compared to lynx [63].

Contrary to our predictions, predation did not appear to take place in locations with less structural cover within a given habitat during most of the year, indicating that hares are vulnerable to predators in a wide variety of habitats regardless of the density of vegetation. However, predation tended to take place in locations with less cover in black spruce and early successional habitats during fall, a time when dispersal by juveniles may have increased their vulnerability to predation when inexperience and poor camouflage already puts them at risk. We could not determine the length of any chases leading to a hare being killed, and it is likely that many hares were seen and pursued by mammalian predators under different cover than where a kill ultimately took place. Nevertheless, most successful chases by lynx and coyotes are <15 m [10], so kills most likely occurred in the same habitat type in which a chase was initiated. Chases are less likely for hawks and owls whose success depends even more on going undetected before making contact with the hare, so the avian kill sites we identified should accurately represent the visibility of hares at the time of detection. It seems feasible that lynx might have benefited from dense vegetation in black spruce forest when stalking and ambushing hares [10]; hunting success depends on getting close to prey without being detected [44] and lynx in Montana killed hares in sites with greater horizontal cover relative to general foraging paths [64]. However, we would not expect kills by goshawks and great horned owls in dense vegetation unless hares were limited to those areas. In general, hares were killed by great horned owls in habitats with less cover, either in shrubs with sparse cover during fall, or in a mixed forest with open understory in other seasons. Previous studies suggest that this is more suitable foraging habitat than black spruce for both great horned owls and goshawks [9, 65], yet over a third of the goshawk predation we identified took place under dense cover in black spruce forest. Hares are clearly vulnerable to predators in every habitat they occupy in the boreal forest, and even dense coniferous forest may not provide significant refuge during times of peak predator numbers.

## Conclusions

Our findings suggest that survival rates of snowshoe hares differ markedly from month to month, and more generally across seasons, depending in part on the habitats hares occupy. Sources of predation also differ considerably as a function of seasonality and habitat, but it is unclear to what extent vegetative cover actually prevents hare predation by specific predators. Despite the protection afforded by vegetation in mature black spruce forests, hares are still highly vulnerable to lynx and goshawk predation in this habitat. Body condition and age influenced survival to a lesser degree in our study, and primarily during fall and winter. The absence of significant decreases in annual survival of older juvenile and adult hares during the population decline suggests that other demographic processes, such as natality and leveret survival, exert important controls over population dynamics [56]. Our observations underscore the temporal variability in snowshoe hare survival and emphasize the importance of a diverse set of biotic variables in controlling this population parameter.

The movement of hares among habitats in our study prevented a clear comparison of habitat-specific survival rates. Identifying the frequency of movements among habitats is important for assessing survival rates of snowshoe hares in patchy landscapes, given that these movements often take place numerous times each day. Obtaining movement data using VHF transmitters can be prohibitively time intensive, so we recommend the use of rapidly advancing GPS technology to more accurately examine habitat-specific survival and movement rates for snowshoe hares. This information may become increasingly important to wildlife management in the boreal forest where a projected increase in wildfires will likely affect habitat patchiness, suitability, and carrying capacities for hares and their predators. Mature black spruce forest might be one of the best refugia for snowshoe hares in Alaska, but due to the flammability of this forest type, hares may face a lack of refugia as wildfires become more frequent. In light of the projected fire regime, we may ultimately see a large-scale decline in carrying capacity for hares and their predators, or a dampening of their population cycles, if hares cannot sustain their numbers in younger habitats.

## Supporting Information

S1 Table Mean ± SE for mass (g), right hind foot length (mm), and body condition index (mass/right hind foot length) for radio-tagged snowshoe hares. (DOCX)

S2 Table Summary of environmental conditions (mean ± SD) in Bonanza Creek Experimental Forest near Fairbanks, Alaska, from June 2008 to May 2012. (DOCX)

S3 Table Candidate models (n = 85) used in known-fate survival analysis of radio-tagged snowshoe hares. Hares were collared in the CONIFER and DECIDUOUS trapping grids in Bonanza Creek Experimental Forest near Fairbanks, Alaska, from June 2008 to May 2012.
(DOCX)
