# Supplementary material for: Seasonal Effects of Habitat on Sources and Rates of Snowshoe Hare Predation in Alaskan Boreal Forests
Source: PLoS One. 2015 Dec 30;10(12):e0143543. doi: 10.1371/journal.pone.0143543 (PMC4696674; doi:10.1371/journal.pone.0143543)
Supplement: S1 Table — (DOCX) [file pone.0143543.s001.docx]

**S1 Table. Mean ± SE for mass (g), right hind foot length (mm), and body condition index (mass/right hind foot length) for radio-tagged snowshoe hares.** Hares were collared in the Conifer and Deciduous trapping grids in Bonanza Creek Experimental Forest near Fairbanks, Alaska, from June 2008 to May 2012.

|  | **n** | **Mass (range)** | **RHF (range)** | **BCI (range)** |
| --- | --- | --- | --- | --- |
| **Adult male** | 105 | 1403 ± 18 (1100–1820) | 138 ± 1 (125–152) | 10.1 ± 0.1 (8.1–12.9) |
| **Adult female** | 125 | 1550 ± 19 (770–2170) | 139 ± 1 (125–151) | 11.1 ± 0.1 (5.5–15.5) |
| **Juvenile male** | 4 | 1287 ± 43 (1190–1370) | 136 ± 1 (133–139) | 9.4 ± 0.2 (8.8–9.9) |
| **Juvenile female** | 24 | 1275 ± 52 (820–1620) | 132 ± 3 (109–150) | 9.5 ± 0.3 (7.5–12.1) |
