# Supplementary material for: Seasonal Effects of Habitat on Sources and Rates of Snowshoe Hare Predation in Alaskan Boreal Forests
Source: PLoS One. 2015 Dec 30;10(12):e0143543. doi: 10.1371/journal.pone.0143543 (PMC4696674; doi:10.1371/journal.pone.0143543)
Supplement: S2 Table — (DOCX) [file pone.0143543.s002.docx]

**S2 Table. Summary of environmental conditions (mean ± SD) in Bonanza Creek Experimental Forest near Fairbanks, Alaska, from June 2008 to May 2012.**

|  | **Avg daily temp (°C)** | **Avg daily min. temp (°C)** | **Avg monthly precip falling as rain (mm)** | **Avg monthly precip falling as snow (mm)** | **Avg daily snow depth (cm)** |
| --- | --- | --- | --- | --- | --- |
| **Jan** | -26 ± 11 | -27 ± 11 | 0 ± 0 | 7 ± 7 | 31 ± 9 |
| **Feb** | -20 ± 10 | -21 ± 10 | 0 ± 0 | 12 ± 11 | 39 ± 11 |
| **Mar** | -16 ± 8 | -17 ± 8 | 0 ± 0 | 12 ± 9 | 48 ± 13 |
| **Apr** | -4 ± 6 | -5 ± 6 | 4 ± 5 | 1 ± 2 | 25 ± 22 |
| **May** | 5 ± 5 | 4 ± 5 | 7 ± 6 | 0 ± 0 | 0 ± 0 |
| **Jun** | 11 ± 3 | 9 ± 3 | 36 ± 12 | 0 ± 0 | 0 ± 0 |
| **Jul** | 13 ± 3 | 11 ± 3 | 38 ± 22 | 0 ± 0 | 0 ± 0 |
| **Aug** | 9 ± 4 | 8 ± 4 | 47 ± 28 | 0 ± 0 | 0 ± 0 |
| **Sep** | 4 ± 5 | 2 ± 5 | 15 ± 6 | 0 ± 0 | 0 ± 0 |
| **Oct** | -7 ± 6 | -8 ± 8 | 1 ± 0 | 10 ± 8 | 4 ± 5 |
| **Nov** | -19 ± 10 | -20 ± 10 | 2 ± 4 | 16 ± 15 | 16 ± 6 |
| **Dec** | -22 ± 10 | -23 ± 10 | 0 ± 1 | 11 ± 7 | 24 ± 7 |
