# Supplementary material for: Seasonal Effects of Habitat on Sources and Rates of Snowshoe Hare Predation in Alaskan Boreal Forests
Source: PLoS One. 2015 Dec 30;10(12):e0143543. doi: 10.1371/journal.pone.0143543 (PMC4696674; doi:10.1371/journal.pone.0143543)
Supplement: S3 Table — Hares were collared in the CONIFER and DECIDUOUS trapping grids in Bonanza Creek Experimental Forest near Fairbanks, Alaska, from June 2008 to May 2012. (DOCX) [file pone.0143543.s003.docx]

**S3 Table. Candidate models (*n* = 85) used in known-fate survival analysis of radio-tagged snowshoe hares.** Hares were collared in the Conifer and Deciduous trapping grids in Bonanza Creek Experimental Forest near Fairbanks, Alaska, from June 2008 to May 2012.

| **Model** | **Parameters** |
| --- | --- |
| S (age) | 2 |
| S (air temperature) | 2 |
| S (body condition) | 2 |
| S (site) | 2 |
| S (snow depth) | 2 |
| S (snow presence) | 2 |
| S (snowfall) | 2 |
| S (age + air temperature) | 3 |
| S (age + snow depth) | 3 |
| S (age + snow presence) | 3 |
| S (age + snowfall) | 3 |
| S (body condition + air temperature) | 3 |
| S (body condition + snow depth) | 3 |
| S (body condition + snow presence) | 3 |
| S (body condition + snowfall) | 3 |
| S (sex) | 3 |
| S (site + age) | 3 |
| S (site + air temperature) | 3 |
| S (site + body condition) | 3 |
| S (site + snow depth) | 3 |
| S (site + snow presence) | 3 |
| S (site + snowfall) | 3 |
| S (snow depth + air temperature) | 3 |
| S (snow presence + air temperature) | 3 |
| S (snowfall + air temperature) | 3 |
| S (age + snow depth + air temperature) | 4 |
| S (age + snow presence + air temperature) | 4 |
| S (age + snowfall + air temperature) | 4 |
| S (body condition * air temperature) | 4 |
| S (body condition + snow depth + air temperature) | 4 |
| S (body condition + snow presence + air temperature) | 4 |
| S (body condition + snowfall + air temperature) | 4 |
| S (season) | 4 |
| S (sex + air temperature) | 4 |
| S (sex + snow depth) | 4 |
| S (sex + snow presence) | 4 |
| S (sex + snowfall) | 4 |
| S (site * age) | 4 |
| S (site * air temperature) | 4 |
| S (site * snow depth) | 4 |
| S (site * snow presence) | 4 |
| S (site + air temperature + age) | 4 |
| S (site + air temperature + snow depth) | 4 |
| S (site + air temperature + snow presence) | 4 |
| S (site + body condition + air temperature) | 4 |
| S (site + body condition + snow depth) | 4 |
| S (site + body condition + snow presence) | 4 |
| S (site + body condition + snowfall) | 4 |
| S (site + sex) | 4 |
| S (site + snow depth + age) | 4 |
| S (site + snow presence + age) | 4 |
| S (site + snowfall + age) | 4 |
| S (site + snowfall + air temperature) | 4 |
| S (snowfall * air temperature) | 4 |
| S (year) | 4 |
| S (age + season) | 5 |
| S (age + year) | 5 |
| S (body condition + season) | 5 |
| S (body condition + year) | 5 |
| S (site + season) | 5 |
| S (site + year) | 5 |
| S (sex + season) | 6 |
| S (sex + year) | 6 |
| S (site * sex) | 6 |
| S (site + body condition + season) | 6 |
| S (site + body condition + year) | 6 |
| S (site + season + age) | 6 |
| S (site + year + age) | 6 |
| S (age * season) | 8 |
| S (age * year) | 8 |
| S (site * season) | 8 |
| S (site * year) | 8 |
| S (sex * year) | 11 |
| S (month) | 12 |
| S (sex * season) | 12 |
| S (age + month) | 13 |
| S (body condition + month) | 13 |
| S (site + month) | 13 |
| S (sex + month) | 14 |
| S (site + body condition + month) | 14 |
| S (site + month + age) | 14 |
| S (age * month) | 23 |
| S (site * month) | 24 |
| S (sex * month) | 36 |
| S (time) | 48 |
